# Brussowvirus SW13 Requires a Cell Surface-Associated Polysaccharide To Recognize Its Streptococcus thermophilus Host

**DOI:** 10.1128/AEM.01723-21

**Published:** 2022-01-11

**Authors:** Katherine Lavelle, Irina Sadovskaya, Evgeny Vinogradov, Philip Kelleher, Gabriele A. Lugli, Marco Ventura, Douwe van Sinderen, Jennifer Mahony

**Affiliations:** a School of Microbiology, University College Cork, Cork, Ireland; b APC Microbiome Ireland, University College Cork, Cork, Ireland; c Université Littoral Côte d’Opale, UMR 1158 BioEcoAgro, Institut Charles Viollette, USC ANSES, INRAE, Université Artois, Université Lille, Université Picardie Jules Verne, Université Liège, Junia, Boulogne-sur-Mer, France; d National Research Council Canada, Ottawa, Ontario, Canada; e Laboratory of Probiogenomics, Department of Chemistry, Life Sciences and Environmental Sustainability, University of Parma, Parma, Italy; f Microbiome Research Hub, University of Parma, Parma, Italy; University of Tennessee at Knoxville

**Keywords:** bacteriophage, dairy fermentation, rhamnose-glucose polysaccharide, cell wall structure, bacteriophage receptor, glycosyltransferase

## Abstract

Four bacteriophage-insensitive mutants (BIMs) of the dairy starter bacterium Streptococcus thermophilus UCCSt50 were isolated following challenge with Brussowvirus SW13. The BIMs displayed an altered sedimentation phenotype. Whole-genome sequencing and comparative genomic analysis of the BIMs uncovered mutations within a family 2 glycosyltransferase-encoding gene (*orf*06955_UCCSt50_) located within the variable region of the cell wall-associated rhamnose-glucose polymer (Rgp) biosynthesis locus (designated the *rgp* gene cluster here). Complementation of a representative BIM, S. thermophilus B1, with native *orf*06955_UCCSt50_ restored phage sensitivity comparable to that of the parent strain. Detailed bioinformatic analysis of the gene product of *orf*06955_UCCSt50_ identified it as a functional homolog of the Lactococcus lactis polysaccharide pellicle (PSP) initiator WpsA. Biochemical analysis of cell wall fractions of strains UCCSt50 and B1 determined that mutations within *orf*06955_UCCSt50_ result in the loss of the side chain decoration from the Rgp backbone structure. Furthermore, it was demonstrated that the intact Rgp structure incorporating the side chain structure is essential for phage binding through fluorescence labeling studies. Overall, this study confirms that the *rgp* gene cluster of S. thermophilus encodes the biosynthetic machinery for a cell surface-associated polysaccharide that is essential for binding and subsequent infection by Brussowviruses, thus enhancing our understanding of S. thermophilus phage-host dynamics.

**IMPORTANCE** Streptococcus thermophilus is an important starter culture bacterium in global dairy fermentation processes, where it is used for the production of various cheeses and yogurt. Bacteriophage predation of the species can result in substandard product quality and, in rare cases, complete fermentation collapse. To mitigate these risks, it is necessary to understand the phage-host interaction process, which commences with the recognition of, and adsorption to, specific host-encoded cell surface receptors by bacteriophage(s). As new groups of S. thermophilus phages are being discovered, the importance of underpinning the genomic elements that specify the surface receptor(s) is apparent. Our research identifies a single gene that is critical for the biosynthesis of a saccharidic moiety required for phage adsorption to its S. thermophilus host. The acquired knowledge provides novel insights into phage-host interactions for this economically important starter species.

## INTRODUCTION

Streptococcus thermophilus belongs to the lactic acid bacteria (LAB) and is utilized very extensively as a starter culture in both industrial and artisanal dairy fermentations. Regressive evolution and gene decay (1) within S. thermophilus coupled with an adaptation to the dairy niche (2) have rendered S. thermophilus the only member of the streptococci to be assigned a generally regarded as safe (GRAS)/qualified presumption of safety status (3, 4). Certain metabolic products of S. thermophilus, which include lactic acid, exopolysaccharide (EPS), and volatile aromatic compounds, impart desirable organoleptic and rheological properties to both yogurt and hard cheeses. Fermentation failure may lead to substantial economic losses due to raw ingredient wastage and substandard products, which negatively impact consumer confidence. Such fermentation failures and inconsistencies are often the result of predation by lytic bacteriophages. To date, five groups of S. thermophilus phages have been characterized: *Moineauvirus* (formerly termed *cos*), *Brussowvirus* (formerly termed *pac*), 5093, 987, and P738 (5–9). Phage predation is highly problematic for S. thermophilus in dairy fermentations, yet despite decades of research, little is known about the host-encoded phage receptors used by these destructive parasites. Such knowledge is essential to facilitate the development of rational starter culture rotation schemes and, in doing so, the incorporation of barriers to phage proliferation. Brussowviruses represent the second most problematic dairy streptococcal phage species and can provide unique challenges since this group incorporates temperate members. For example, the integration of temperate phage Φ20617 within the host genome results in compromised cell wall integrity, heat resistance, and increased surface adhesion (10, 11).

The immunity of S. thermophilus to its phages appears to be primarily driven by the activity of its encoded CRISPR-Cas systems (12), although receptor modification may also represent an important source of phage resistance development. Recently, exopolysaccharide has been confirmed as the primary receptor for the 987 phage group (13), and several studies have confirmed that cell surface-associated polysaccharides, including those specified by the rhamnose-glucose polymer (*rgp*) and exopolysaccharide (*eps*) loci, are required for infection by the prolific *Moineauvirus* and *Brussowvirus* species (14–17). Unlike other ovococcal LAB species, significant knowledge gaps surround the physiological function of the rhamnose-containing cell wall polysaccharide of S. thermophilus and its role in the initial stages of phage infection. The dynamic interaction between S. thermophilus phages and host-encoded cell surface polysaccharides is further complicated by the presence of multiple and variable carbohydrate binding domains (CBDs) encoded by the relevant phage genome region presumed to specify host recognition functions (18–20).

To date, only a single representative of S. thermophilus Rgp (that of St64987) has been elucidated, revealing a complex structure comprised of a linear rhamnan core with tri- and tetrasaccharide decorations (13). In this study, we present the second known biochemical structure of a rhamnose-containing cell wall polysaccharide from S. thermophilus, which displays significant variance from that of St64987. Furthermore, we experimentally ascertain that the *rgp* locus of S. thermophilus encodes the biosynthetic functions required for the production of cell surface-associated saccharidic moieties, which are essential for a Brussowvirus-type phage infection.

## RESULTS

### BIM isolation and *in silico* characterization.

While DT1 (21, 22) and STP1 (18) have become useful prototypic Moineauviruses to evaluate phage-host interactions, limited studies pertaining to Brussowviruses are available. To bridge this knowledge gap, the Brussowvirus host strain UCCSt50::pNZ44-acrIIA6 was challenged with Brussowvirus SW13 (10^7^ PFU mL^−1^). Surviving colonies were picked and assessed for phenotypic changes in broth (defined here as heavy sedimentation) following overnight incubation and phage resistance, which resulted in the generation of four stable bacteriophage-insensitive mutants (BIMs) (named B1, B2, B4, and B9), which were selected for detailed investigation.

All BIMs displayed a phage-insensitive profile against SW13 and a heavy-sedimentation phenotype in broth cultures compared to those of the parental strain, which displays a homogeneous growth phenotype and moderate sedimentation. To obtain genetic insights into the basis of their acquired phage insensitivity, the genomes of SW13-resistant BIMs B1, B2, B4, and B9 were sequenced. Analysis of the CRISPR1 and CRISPR3 arrays of each BIM confirmed that the resistance profile was not CRISPR mediated (see Table S3 in the supplemental material). Alterations within cell wall-associated glycosyltransferases (GTs) have previously been shown to induce a sedimenting phenotype in broth in addition to morphological defects. Such alterations may also lead to phage insensitivity due to the abolition of the host-encoded cell surface receptor (13, 23–27).

Comparative genomic analysis of the four BIMs versus the parent strain using SNP (single nucleotide polymorphism) analysis identified between two and five genes harboring SNPs of 100% allelic variation (Table 1).

**TABLE 1:** Summary of the SNPs identified across B1, B2, B4, and B9 with 100% allelic variation compared to the parent strain UCCSt50^*a*^

Strain and SNP position	Outcome	Location
B1		
247848	A^190^-V^190^	MurC
497603	D^11^-N^11^	Nucleotidyltransferase
521615	F^25^-V^25^	ABC transporter, ATP binding
**1339893**	**Q^170^-stop**	**Glycosyltransferase**
1652118	F^100^-F^100^	Queuine tRNA ribosyltransferase

B2		
**1340323**	**Y^26^-stop**	**Glycosyltransferase**
1652118	F^100^-F^100^	Queuine tRNA ribosyltransferase

B4		
67110	C^606^-Y^606^	DNA mismatch repair protein
334432	V^911^-A^911^	Translation initiation factor IF-2
**1340051**	**G^117^-D^117^**	**Glycosyltransferase**
1652118	F^100^-F^100^	Queuine tRNA ribosyltransferase

B9		
**1340045**	**R^119^-H^119^**	**Glycosyltransferase**
1652118	F^100^-F^100^	Queuine tRNA ribosyltransferase

a Only those SNPs located within *orf*06955_UCCSt50_ (boldface type) displayed variation between each BIM.

All BIMs were found to harbor a synonymous SNP at position 1652118 (*orf*8525). As the primary amino acid sequence was identical to that of the parent strain, this genomic variance was omitted from further investigative analysis.

**FIG 1:**
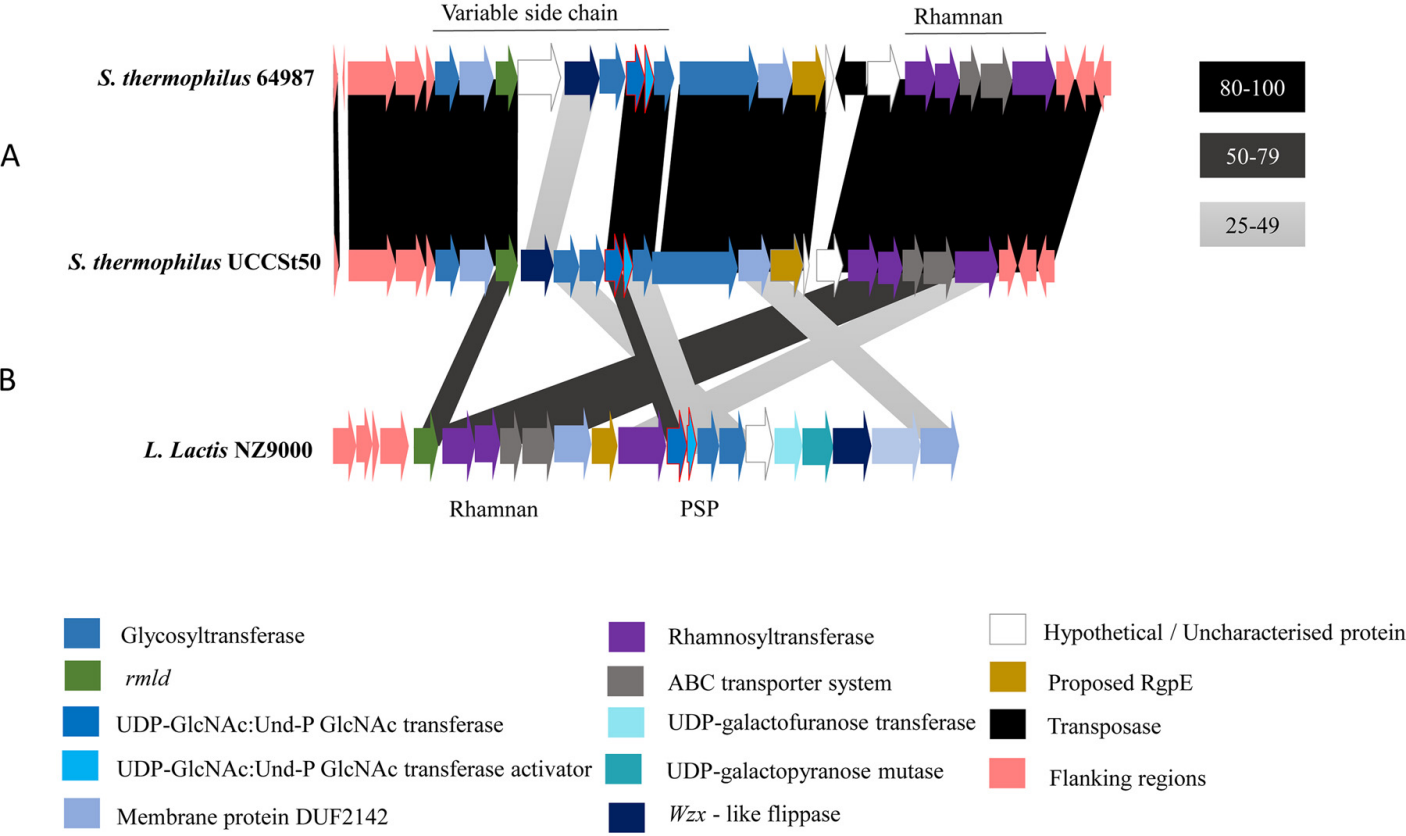
Overall genome arrangement of the S. thermophilus UCCSt50 *rgp* cluster. The two genes encoding the GacI/J-like proteins are outlined in red. (A) Comparative analysis between the *rgp* clusters of UCCSt50 and ST64987 showing significant variation in the region associated with the biosynthesis of the decorative side chain. (B) Comparative analysis between the *rgp* loci of S. thermophilus strains UCCSt50 and ST64987 and the *cwps* cluster of L. lactis NZ9000 indicating the detected homology to WpsA and the gene products responsible for rhamnan biosynthesis.

Most notably, each BIM was found to harbor a distinct mutation within *orf*06955, which encodes a predicted glycosyltransferase within the *rgp* cluster of this strain’s genome (Fig. 1). In the case of B1 and B2, the mutations at the respective positions 1339893 and 1340323 lead to the incorporation of stop codons replacing glutamine (Q170) and tyrosine (Y26), thus resulting in the truncation of the encoded protein. For BIMs B4 and B9, the mutations in *orf*06955 are located at positions 1340051 and 1340045, causing amino acid substitutions G117D and R119H, respectively. Since this gene, *orf*06955_UCCSt50_, was mutated in all four BIMs, and the predicted function of its encoded product is as a glycosyltransferase associated with cell wall polysaccharide biosynthesis, it was selected for further investigation and complementation studies to assess its function in the observed SW13 phage resistance phenotype.

### *orf*06955_UCCSt50_ is involved in SW13-host interactions.

To confirm that mutations within *orf*06955_UCCSt50_ are indeed responsible for the acquired SW13 resistance phenotype, the native *orf*06955_UCCSt50_ was cloned into pNZ44-acrIIA6 and transformed into the representative mutant B1 to generate strain B1::pNZ44-06955. The sensitivity of the latter strain to SW13 was restored to the same order of magnitude (efficiency of plaquing [EOP] = 0.59) as that of UCCSt50 (Table 2), confirming that *orf*06955_UCCSt50_ is directly involved in the infection process of SW13. The EOP of SW13 on BIMs B2, B4, and B9 in addition to B1ΔpNZ44-06955, a strain derived from strain B1::pNZ44-06955 from which plasmid pNZ44-06955 was cured, was also determined (and calculated relative to that of UCCSt50), revealing values similar to those observed for strain B1 (Table 2).

**TABLE 2:** Relative EOPs of phage SW13 on its primary host UCCSt50 and its derived BIMs^*a*^

Strain	EOP of SW13
S. thermophilus UCCSt50	1
S. thermophilus B1	≤2.21 × 10^−7^
S. thermophilus B2	≤2.21 × 10^−7^
S. thermophilus B4	≤2.21 × 10^−7^
S. thermophilus B9	≤2.21 × 10^−7^
S. thermophilus B1::pNZ44-acrIIA6	≤2.21 × 10^−7^
S. thermophilus B1::pNZ44-06955	5.9 × 10^−1^ ± 4.10 × 10^−1^
S. thermophilus B1ΔpNZ44-06955	≤2.21 × 10^−7^

a Complementation of B1 with native *orf*06955_UCCSt50_ restored sensitivity to the same order as that of the parent strain.

### *orf*06955_UCCSt50_ encodes a proposed initiator of polysaccharide side chain biosynthesis in UCCSt50.

Initial characterization of ORF06955_UCCSt50_ was performed using BLASTP and HHpred. Both outputs revealed significant similarities (≥95%) to family 2 glycosyltransferases involved in cell wall biosynthesis. No transmembrane domains were detected using the TMHMM server, suggesting a possible cytosolic localization of ORF06955_UCCSt50_. In order to assign a putative function, the overall bioinformatic output of ORF06955_UCCSt50_ was combined and compared by BLASTP to the proteins harboring similar topologies and of the same GT family (gene products of *llnz_1135*, *llnz_1145*, and *llnz_1150*) encoded by the variable polysaccharide pellicle (PSP) region of the Lactococcus lactis NZ9000 *cwps* cluster, the encoded functions of which have recently been described in detail (27). This analysis revealed that ORF06955_UCCSt50_ shares significant identity (51.46% amino acid identity with 100% query coverage) with WpsA (encoded by *llnz_1135* [*wpsA*]) of Lactococcus lactis NZ9000, a 245-amino-acid (aa) GT that is presumed to initiate PSP synthesis through the transfer of GlcNAc from UDP-GlcNAc to the lipid carrier undecaprenyl phosphate (Und-P) (27) (Fig. 1). WpsA is a functional equivalent of GacI, the well-characterized UDP-GlcNAc:Und-P GlcNAc transferase of Streptococcus pyogenes, which is involved in GlcNAc modification of the rhamnan backbone (28), yet WpsA displays limited identity to GacI (43% at the amino acid level) (27). Homologs of GacI have also been identified in other species, including various streptococci (including a representative strain of S. thermophilus) and enterococci (28, 29). Based on the observed similarities, we propose that ORF06955_UCCSt50_ represents a UDP-GlcNAc:Und-P GlcNAc transferase, which is involved in the formation of a cell surface-associated polysaccharide that is essential for SW13 infection.

### Biochemical investigation of the Rgp structure of UCCSt50.

To confirm if mutations within *orf*06955_UCCSt50_ affect the cell wall-associated polysaccharide (CWPS) structure (as observed for genes encoding the GacI-like proteins of L. lactis, S. pyogenes, and Enterococcus faecalis), a detailed biochemical investigation was performed to analyze the Rgp structure of both the parent strain UCCSt50 and a representative BIM, B1 (which was randomly selected). The Rgp preparation of wild-type (WT) strain UCCSt50 contained Rha, Glc, Gal, and GlcNAc in an approximate ratio of 2.8:1:0.8:0.7 (detector response). Nuclear magnetic resonance (NMR) spectra and methylation profiles indicated a complex branched structure containing 3-linked GlcNAc.

**FIG 2:**
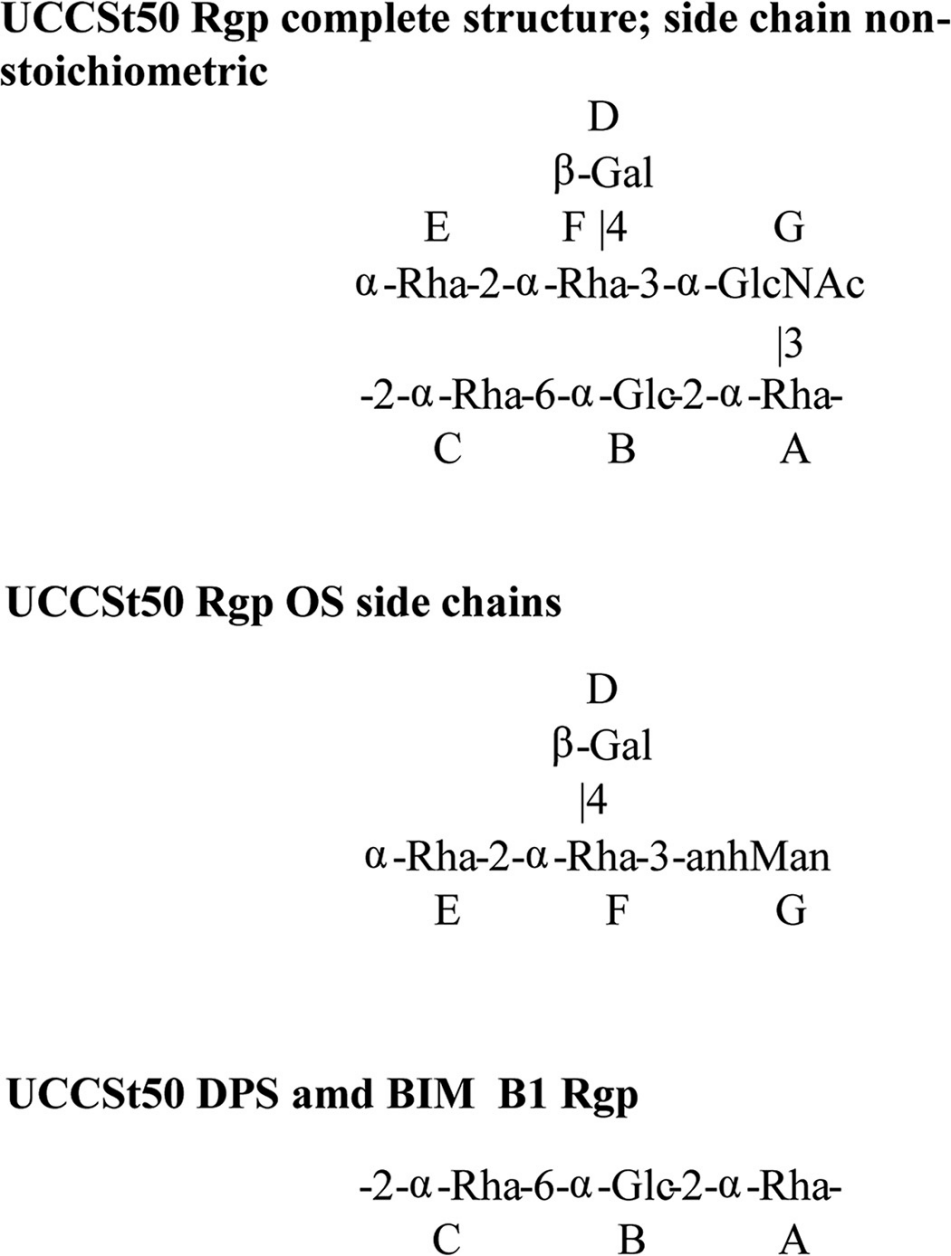
Structures of the Rgp from S. thermophilus strain UCCSt50, oligosaccharide (OS) and polysaccharide (DPS) products of its deamination, and Rgp of its mutant B1.

**FIG 3:**
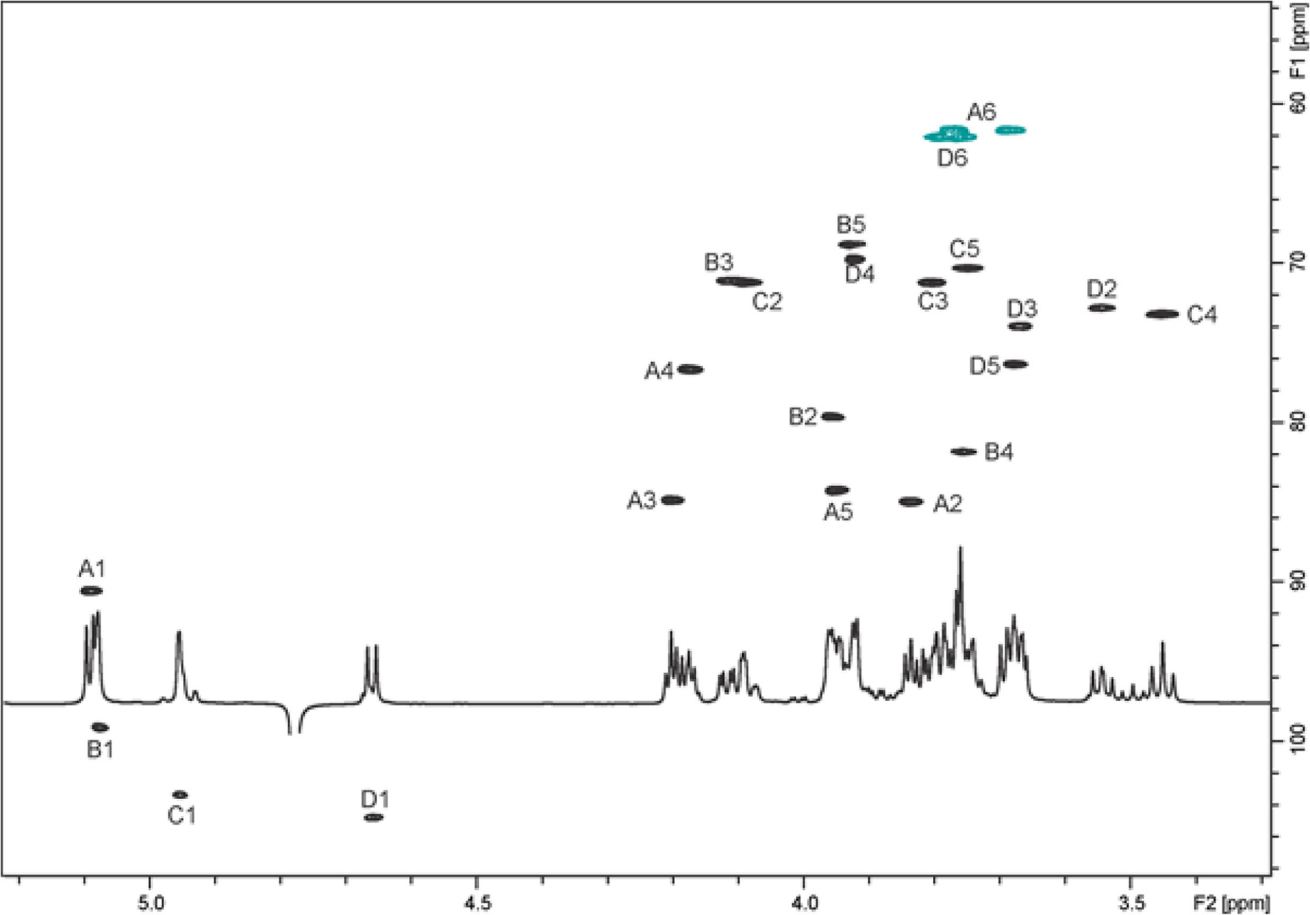
^1^H-^13^C heteronuclear single quantum coherence (HSQC) spectrum of the S. thermophilus UCCSt50 deaminated OS.

The Rgp of UCCSt50 was subjected to deacetylation and deamination to selectively cleave the sugar chain at the GlcNAc residue and obtain fragments of Rgp (13, 30). This treatment afforded deaminated polysaccharide (DPS) and oligosaccharide (OS) fractions, which were analyzed by two-dimensional (2D) NMR. Complete assignment of the 2D NMR spectra and analysis of nuclear Overhauser effect spectroscopy (NOESY) of the DPS led to the identification of a linear trisaccharide-repeating unit containing two 2-linked α-Rha residues and a 6-linked α-Glc residue (Fig. 2; Table S4), identical to the linear backbone of the previously described Rgp from St64987 (13). 2D NMR analysis of the purified OS fraction allowed us to identify a branched tetrasaccharide OS with a 3-substituted 2,5-anhydro-mannose (product of the deamination of glucosamine) at the reducing end (Fig. 2 and 3; Table S4). These data, taken together, indicate that S. thermophilus UCCSt50 Rgp is composed of a backbone polymer made up of trisaccharide-repeating units and carrying tetrasaccharide side chains with GlcNAc at branching points.

Detailed 2D NMR analysis of the Rgp of strain UCCSt50 (Fig. S1 and Table S4) allowed the elucidation of the complete structure of this polymer and showed a backbone composed of trisaccharide-repeating units (–2-α-Rha-6-α-Glc-2-α-Rha–), which carry a tetrasaccharide side chain on the majority of Rha residues (Fig. 2). Methylation analysis confirmed the structure established by NMR and showed the presence of terminal and 2-, 2,3-, and 2,4-linked Rha; 6-linked Glc; 3-linked GlcNAc; and terminal Gal.

2D NMR analysis of the Rgp of the mutant strain B1 showed a linear polysaccharide identical to the −2-α-Rha-6-α-Glc-2-α-Rha– trisaccharide-repeating backbone of the Rgp of the parent strain UCCSt50; however, this linear polysaccharide lacks side chains (Fig. 2; Fig. S2).

A structural analysis of the genetically complemented strain, i.e., B1::pNZ44-06955, confirmed the restoration of the branched side chain structure on the Rgp polymeric backbone. Therefore, we conclude that *orf*06955_UCCSt50_ is a functional homolog of WpsA/GacI and that the mutation present in strain B1 (and probably in the other BIMs) prevents side chain modification of the Rgp polymeric core, which in turn results in resistance to phage SW13.

### The tetrasaccharide side chain of the Rgp of S. thermophilus UCCSt50 is essential for SW13 host binding.

Alterations to the rhamnose-containing cell wall polysaccharide structure(s) of L. lactis are known to induce a strong phage resistance phenotype due to a lack of adsorption at the cell surface (27). To establish if the same is true for alterations to the Rgp of S. thermophilus, traditional adsorption assays of phage SW13 on its host UCCSt50 and the Rgp mutant B1 were undertaken However, adsorption to UCCSt50 was found to be unstable (calculated as 74.48% ± 11.64%), indicating that this method would be inappropriate for the assessment of potential adsorption deficiencies induced by mutations within *orf*06955. Therefore, alternative methods to determine if the adsorption of phage SW13 to its host was impeded by the removal of the Rgp branched side chain were explored. We recently characterized the putative receptor binding protein (RBP) CBD of the *Moineauvirus* phage STP1 (18) as a functional, host-specific binding region that shares strong structural homology to the RBPs of lactococcal phages TP901-1 and p2 (31, 32) and *Listeria* phage PSA (33). This domain was detected across all *Moineauvirus* and *Brussowvirus* members of S. thermophilus, including phage SW13. It was therefore decided to clone the region encoding the CBD of the putative RBP of phage SW13 into pHPT9 to allow the heterologous production of recombinant green fluorescent protein (Gfp)-RBP-module_SW13_ in order to assess the impact of mutations within *orf*06955_UCCSt50_ on the phage-host interaction process.

**FIG 4:**
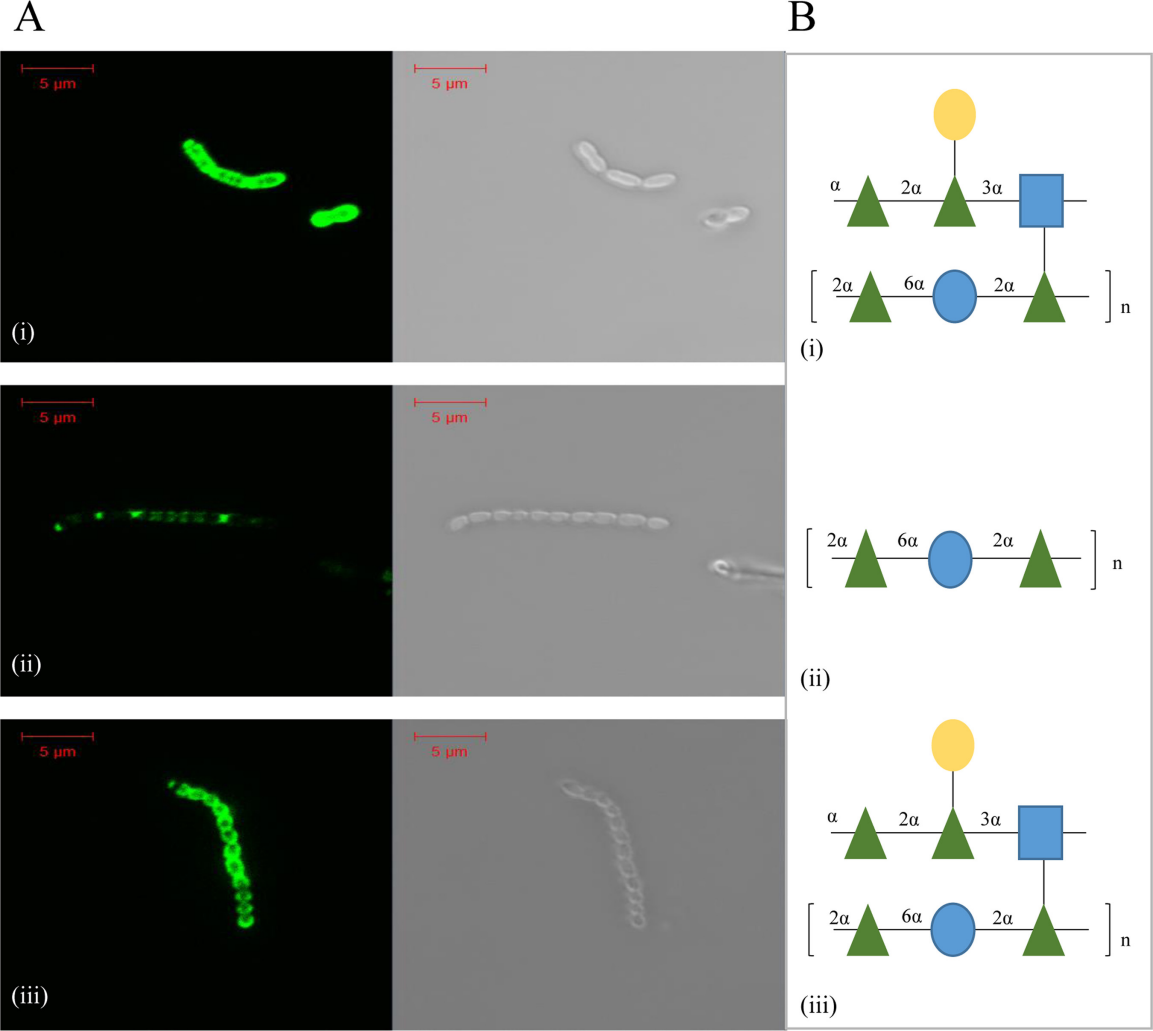
(A) Fluorescence labeling (Gfp excitation wavelength of 488 nm) of parent strain S. thermophilus UCCSt50 (i), its phage-resistant derivative B1 (ii), and the genetically complemented strain B1::pNZ44-06955 (iii) using 5 μg protein. (B) Associated biochemical structure of the Rgp isolated from parent strain UCCSt50 (i), B1 (ii), and B1::pNZ44-06955 (iii).

Fluorescence labeling assays using 5 μg Gfp-RBP-module_SW13_ showed complete cell surface labeling of the parent strain UCCSt50, which possesses an intact branched Rgp polysaccharide (Fig. 4A). In contrast, labeling of the derivative B1, which lacks the branched element of the Rgp polysaccharide (Fig. 4B), was partial. Labeling of B1 with Gfp-RBP-module_SW13_ was quantified at 2.93% ± 0.55% (Table 3), relative to that of UCCSt50 (normalized to 100%).

**TABLE 3:** Quantification of the adsorption of Gfp-RBP-module_SW13_ to B1 and B1::pNZ44-06955 relative to that of UCCSt50 using a Qubit fluorometer^*a*^

Strain	Avg % fluorescence ± SD
S. thermophilus UCCSt50	100
S. thermophilus B1	2.93 ± 0.55
S. thermophilus B1::pNZ44-06955	76.59 ± 6.3

a Percentages shown represent the averages from three replicate assays ± standard deviations.

To confirm that the mutations of *orf*06955_UCCSt50_ were solely responsible for the >30-fold-reduced binding ability observed for B1, its complemented derivative B1::pNZ44-06955 was also subjected to labeling assays. As shown in Fig. 4Aiii, the binding of the complemented derivative was comparable to that of the parent strain and quantified at 76.59% ± 6.3%. The binding of Gfp-RBP-module_SW13_ to the complemented derivative B1::pNZ44-06955, compared to that of B1, was found to be statistically significant (*P* ≤ 0.0001).

Overall, these results confirm that efficient binding of Gfp-RBP-module_SW13_ (and, by inference, phage infection [Table 3]) to its host is dependent on the presence of a complete, branched Rgp structure on the cell surface.

## DISCUSSION

The exact nature of the host-encoded receptor of S. thermophilus phages has until recently been poorly defined, and only a small number of studies have been undertaken to address this knowledge gap (16, 17). Recently, EPS has been proven to mediate adsorption by members of the rare 987 phage group (13), while conversely, mutations in genes encoding Rgp biosynthesis-associated GTs have been correlated with phage resistance (14, 15). In the current study, we isolated four derivatives of UCCSt50 that are resistant to Brussowvirus SW13 and showed that they all harbor mutations in *orf*06955_UCCSt50_, a GT-encoding gene of the *rgp* cluster that appears to represent a GacI/WpsA homolog.

The variable region of the polyrhamnose structures in these species has previously been shown to be important for phage infection. The serospecific region of the *rgp* cluster of S. mutans has long been established as being critical for phage adsorption (34), and mutations in the enterococcal polysaccharide antigen (EPA) cluster in E. faecalis directly affect host interactions for phage ΦNPV1 (26). In the case of L. lactis, the *cwps* clusters can be correlated with the phage host range and receptor binding phylogeny of the prolific 936 phage group (skunaviruses) (24, 35, 36), while recent in-depth studies of PSP mutant derivatives of L. lactis NZ9000 have further defined the roles of *wpsA* to *wpsF* (*wpsA*–*F*) and *wpsH*–*J* during early infection by phages p2, sk1, and jj50 (27). Szymczak et al. recently provided evidence that links phage infection ability to the variable region of the *rgp* cluster in S. thermophilus through the isolation of a phage-resistant variant that harbored mutations in GTs of the EPS and *rgp* clusters and was adsorption deficient for the Brussowvirus phage CHPC1057 (14, 15). The present study not only corroborates these findings but also provides an in-depth view into the role of the *rgp* cluster variable region of S. thermophilus in phage-host interactions through a combination of comparative genome and Rgp analysis, complementation, and binding assays of the phage-resistant mutants. The critical role of UDP:Und-P transferases in phage infection has also been characterized in the Gram-negative pathogen Klebsiella pneumoniae. Tan et al. (37) observed that the insertion of mobile genetic elements within *wcaJ*, which encodes a UDP-glucose:Und-P glucose-1-phosphate transferase, induces strong resistance to the *Podoviridae* phage 117 (37), while a study by Cai et al. (38) determined that the downregulation of WcaJ and two additional capsular polysaccharide GTs, GT-1 and GT-2, in K. pneumoniae K7 leads to resistance against siphophage GH-K3. Interestingly, it was found that the adsorption efficiency of GH-K3 could be significantly improved (48.9% to 84.2%) in the Δ*wcaJ* and Δ*GT-1* backgrounds by increasing the incubation times to 1 h, indicating that the accumulation of macromolecular CWPSs in these mutants is sufficient for phage binding. A similar pattern of phage-host interaction was noted for L. lactis *cwps* mutants whereby phage escape mutants could be isolated on host derivatives that produced residual or truncated PSP (27). A definitive outcome could not be established for phage SW13 and *orf*06955_UCCSt50_ mutants using standard adsorption assays. However, fluorescence labeling assays confirmed that the loss of the decorative, branched structure from the core rhamnan polysaccharide results in significantly reduced phage receptor binding, and quantification of the fluorescence confirmed that levels on B1 are in fact in line with what is classified as adsorption deficient in the literature with respect to surface glycan mutants (13, 39).

The outcomes of this study not only confirmed that the host-encoded variable region of the *rgp* cluster is essential for SW13 phage infection of S. thermophilus UCCSt50 but also provided insights into the biosynthesis of this physiologically important polymer. Furthermore, we present the second known biochemical structure of an S. thermophilus Rgp, which displays significant variance in the side chain structure compared to that of the previously elucidated polymer of St64987 (13). This observed variability is in line with current knowledge on S. thermophilus *rgp* clusters. In 2019, it was proposed that S. thermophilus strains can be classified into at least five *rgp* genotypes, Rgp type A through Rgp type E, based on phylogenetic analysis (15). This was further refined in 2020 where it was proposed that Rgp types A, B, and C comprise the three main groups, with the previously independent groups D and E becoming subsets of types A and C, respectively (40). Such high levels of diversity at the genomic level suggest that S. thermophilus may encode multiple side chain structures. Similar to recent findings in L. lactis, further investigative studies on the biochemical structures of additional S. thermophilus strains may allow for a correlation between the Rgp genotype and the chemical composition of their associated Rgp.

## MATERIALS AND METHODS

### Bacterial strains and bacteriophages used in this study.

S. thermophilus UCCSt50 and its derivatives (see Table S1 in the supplemental material) were routinely grown from a single colony or bacterial stocks maintained at −20°C (25% [wt/vol] glycerol) in M17 broth (Sigma, USA) supplemented with 0.5% lactose at 42°C. For S. thermophilus strains UCCSt50::pNZ44-acrIIA6 and B1::pNZ44-06955, chloramphenicol (Sigma, USA) was added to growth medium at a final concentration of 5 μg mL^−1^.

### Bacteriophage assays.

Phage SW13 was propagated as previously described (41) and enumerated using the standard overlay method (42), in which LM17 medium was supplemented with 10 mM CaCl_2_, 0.25% glycine, and 10 g L^−1^ (solid) or 4 g L^−1^ (semisolid) agar-agar (Neogen, USA). Adsorption assays were performed as previously described (13).

### Bacteriophage-insensitive mutant isolation.

Competent cells of S. thermophilus UCCSt50 were prepared as previously described (43), with the following modification: 1% threonine was used in place of glycine. Fifty microliters of cells was mixed with 5 μL of fresh pNZ44-acrIIA6 plasmid DNA (13), transferred to a prechilled electroporation cuvette, and electroporated at 2 kV with subsequent recovery at 42°C for 4 h in HJL medium (3% tryptone, 1% yeast extract, 0.5% KH_2_PO_4_, 0.2% beef extract, 0.5% lactose) (44, 45) supplemented with 20 mM CaCl_2_ and 200 mM MgCl_2_. Subsequently, the transformation mixture was plated onto LM17 agar supplemented with 5 μg mL^−1^ chloramphenicol and incubated for 72 h at 42°C. The resulting strain, designated S. thermophilus UCCSt50::pNZ44-acrIIA6 here, was challenged with its infecting phage SW13 to generate non-CRISPR-mediated bacteriophage-insensitive mutants (BIMs). Briefly, 10 μL of the SW13 phage lysate (≥10^7^ PFU mL^−1^) and 400 μL of a fresh culture of UCCSt50::pNZ44-acrIIA6 grown overnight were mixed in 4 mL of semisolid LM17 agar supplemented with 10 mM CaCl_2_, poured onto LM17 agar plates supplemented with 10 mM CaCl_2_, and incubated at 42°C for 24 to 48 h. Surviving colonies were picked and assessed for insensitivity to phage SW13 using the standard overlay method (as described above). In addition, all BIMs were assessed for their phenotype in broth, compared to that of the parent strain, following growth overnight.

### DNA preparation, genome sequencing, and *in silico* analysis of S. thermophilus strains.

Having established a stable phage insensitivity profile in addition to an altered growth phenotype, four BIMs, designated B1, B2, B4, and B9, were selected for further comparative studies against the parent strain UCCSt50. Genomic DNA of strain UCCSt50 and the four selected BIMs was extracted using the PureLink genomic DNA minikit (Invitrogen), with the following modifications: cells were harvested, and the pellet was resuspended in 200 μL TESL (Tris-EDTA [TE] supplemented with 25% sucrose and 30 mg mL^−1^ lysozyme) and incubated at 37°C for 30 min prior to the kit purification protocols mentioned above. For single-molecule real-time (SMRT) sequencing, the genomic DNA of UCCSt50 was extracted using Macherey-Nagel Nucleobond buffer set III and Nucleobond AXG 100 Midi columns (Macherey-Nagel, Germany) according to the manufacturer’s instructions.

Whole-genome sequencing of the parent strain UCCSt50 was performed with the Illumina MiSeq sequencing system (Genprobio, Parma, Italy), using paired-end reads (2 by 250 bp) in combination with (SMRT) sequencing via a Pacific Biosciences RS II sequencing platform (Macrogen, South Korea). *De novo* assembly of UCCSt50 was performed using the Pacific Biosciences SMRTPortal analysis platform (version 2.3.1) and the RS_HGAP_Assembly.2 protocol. Illumina assembly of UCCSt50 reads was performed with SPAdes v3.12.0 via the MEGAnnotator pipeline (46), and open reading frames (ORFs) were predicted using Prodigal v2.6 (47). Automatic annotations were performed using RAPSearch2 against the HMMER, NCBI, and Pfam databases. rRNA and tRNA gene predictions were performed using RNAmmer v1.2 and tRNAscan SE v1.21, respectively (48, 49). Further manual annotations and functional predictions of ORFs associated with the *rgp* cluster were performed using a combination of BLASTP, Pfam, and HHpred analyses (50, 51). The four BIMs, B1, B2, B4, and B9, were sequenced using Illumina platforms, as described above. A single nucleotide polymorphism (SNP) analysis of B1, B2, B4, and B9 genome sequences against the genome of the parent strain UCCSt50 was performed using the Bowtie2 alignment tool (52) and SAMtools (53).

### BIM complementation.

Native *orf*06955_UCCSt50_ was amplified with HF Phusion polymerase (New England BioLabs, USA) using primer combination 06955F and 06955R (Table S2) and purified using the GenElute PCR cleanup kit (Sigma-Aldrich, UK). Both the *orf*06955_UCCSt50_-encompassing amplicon and pNZ44-acrIIA6 were digested with PstI and SpeI (Roche) at 37°C for approximately 3 h. Following digestion, pNZ44-acrIIA6 was further treated with shrimp alkaline phosphatase (New England BioLabs, USA) at 37°C for 30 min, followed by inactivation at 65°C for 5 min. Ligation of the digested amplicon of *orf*06955_UCCSt50_ and pNZ44-acrIIA6 was performed overnight at room temperature using T4 DNA ligase (Promega, UK). Following dialysis, 10 μL of the ligation mixture was added to 50 μL of competent cells of L. lactis NZ9000 in a prechilled electroporation cuvette and subjected to a single pulse at 2.0 kV. A total of 950 μL of recovery medium (M17 broth supplemented with 0.5 M sucrose, 0.25% glucose, 20 mM CaCl_2_, and 200 mM MgCl_2_) was immediately added. The cells were allowed to recover at 30°C for 2.5 h before plating on GM17 agar supplemented with 5 μg mL^−1^ chloramphenicol and incubating the cells overnight at 30°C under microaerophilic conditions. Plasmid DNA was subsequently extracted using the GeneJET plasmid miniprep kit (Thermo Fisher, UK) and stored at 4°C until needed. The integrity of the recombinant plasmid pNZ44-06955 was confirmed by Sanger sequencing (Eurofins Genomics, Germany) using the primers pNZ44F and pNZ44R (Table S2).

Fresh competent cells of S. thermophilus UCCSt50 B1 were prepared as described above for UCCSt50, with the following modification: 0.2 to 0.3% glycine was used in place of 1% threonine. One hundred microliters of cells was subsequently mixed with 10 μL of pNZ44-06955 plasmid DNA or the control vector pNZ44-acrIIA6 and held on ice for 30 min. Transformation was performed by heat shock at 42°C for 45 s, followed by 1 min on ice, before transferring the mixture to a precooled cuvette and applying a single 2.0-kV pulse. A total of 900 μL HJL recovery medium was added, and phenotypic expression was performed at 42°C for 4 h. Cells were plated on LM17 agar supplemented with 5 μg mL^−1^ chloramphenicol and incubated at 42°C for 48 to 72 h under microaerophilic conditions. Chloramphenicol-resistant transformants were verified to contain the desired recombinant plasmid by means of colony-based PCR using the primer combination pNZ44F and pNZ44R.

### Efficiency-of-plaquing determination.

The efficiency of plaquing (EOP) of phage SW13 on the host strain UCCSt50, all four BIMs, the complemented strain B1::pNZ44-06955, and the control strain B1::pNZ44-acrIIA6 was determined using the standard double-layer method (as described above) to evaluate the role of ORF06955_UCCSt50_ in the SW13 phage adsorption/infection process. To validate that the observed effect was due to the introduction of the native gene, the complementing plasmid was cured from S. thermophilus UCCSt50 B1::pNZ44-06955 through serial passaging in the absence of chloramphenicol. The cured derivative, B1ΔpNZ44-06955, was tested for insensitivity to phage SW13 and sensitivity to chloramphenicol. Colony PCR using the above-mentioned pNZ44-specific primers further confirmed the absence of plasmid pNZ44-acrIIA6.

### Recombinant protein production and purification.

The region encoding the carbohydrate binding domain (CBD) of the putative RBP of SW13 (ORF21_SW13_ residues 479 to 681) was amplified from a fresh phage lysate (10^7^ PFU mL^−1^) using the primers listed in Table S2. The resulting amplicon was purified (GenElute PCR cleanup kit; Sigma) and cloned into the green fluorescent protein (GFP) fusion vector pHPT9 (NZYTech, Portugal) according to the manufacturer’s instructions. The ligation mixture was dialyzed against sterile, distilled H_2_O before transformation into Escherichia coli BL21(DE3) using the heat shock method (42°C for 45 s followed by 2 min on ice). Recovery was performed at 37°C for 1 h before plating the cells onto LB agar supplemented with 50 μg mL^−1^ kanamycin and incubating the cells overnight. Confirmed recombinants were verified by Sanger sequencing (Eurofins, Germany).

For targeted protein expression, 100 mL of autoinduction medium (NZYTech, Portugal) supplemented with 1% glycerol and 50 μg/mL kanamycin was inoculated with 1 mL of a fresh culture of E. coli BL21(DE3) harboring pHTP9-RBP-module_SW13_ grown overnight. Incubation was performed with agitation (300 rpm) at 24°C for 24 h. Cells were harvested at 4,696 × *g* for 30 min, followed by resuspension in lysis buffer (50 mM Tris-HCl [pH 7.5], 300 mM NaCl, 5% glycerol, 1% Triton X-100, 30 mM imidazole, 50 mg/mL lysozyme) and freezing at −80°C for a minimum of 24 h. After thawing, the cells were subjected to five cycles of sonication (MSE Soniprep; Sanyo, Japan) at maximum amplitude for 30 s, followed by a 30-s rest. Cellular debris was separated by centrifugation at 20,000 × *g* at 4°C, followed by a second centrifugation at 4,696 × *g* at room temperature to remove the remaining aggregates. Purification was performed using a standard Ni-nitrilotriacetic acid (NTA) agarose column according to the manufacturer’s instructions (Qiagen, UK), with the recombinant protein of interest eluting in the 150 to 200 mM imidazole fractions. Fractions were quantified using the standard Bradford assay (Bio-Rad, UK), dialyzed against protein storage buffer (50 mM CaCl_2_, 50 mM Tris-HCl [pH 7.5], 300 mM NaCl), and stored at 4°C until required.

### Labeling assays.

Labeling assays were performed as previously described, with minor modifications (20, 54). Briefly, 300 μL of cells of UCCSt50, B1, B1::pNZ44-06955, B9, and the negative-control and nonhost strain UCCSt95 was harvested in the early exponential phase at 2,400 × *g* for 5 min and resuspended in 300 μL SM buffer (50 mM Tris-HCl [pH 7.5], 100 mM NaCl, 10 mM MgSO_4_). Five micrograms of protein was added to the cells, and the mixture was incubated at 42°C for 12.5 min. An equal volume of SM buffer (representing 5 μg) was added as a negative control. Cells were then washed three times in 120 μL SM buffer to remove unbound protein. Fluorescence labeling of cells was visualized using an LSM 5 exciter (Zeiss, Germany) with a Gfp excitation wavelength of 488 nm, and images were processed using the Zen 2.3 software package. Quantification of the labeling of Gfp-RBP-module_SW13_ in strain UCCSt50, B1, or B1::pNZ44-06955 was performed using a method adapted from the one described previously by Lizier et al. (55). Briefly, a standard calibration curve was generated using 0, 3, and 6 μg of Gfp-RBP-module_SW13_ on a Qubit fluorometer. Labeling of UCCSt50, B1, and B1::pNZ44-06955 was performed as described above with a final resuspension in 300 μL before cells were subjected to fluorometric measurement. The statistical significance of the labeling of the complemented strain B1::pNZ44-06955 with Gfp-RBP-module_SW13_ compared to that of B1 was calculated using an unpaired *t* test.

### Preparation and structural elucidation of cell wall polysaccharides.

Cells from 4 L of cultures of UCCSt50, the phage insensitive derivative B1, and its complemented derivative B1::pNZ44-06955 grown overnight were harvested by centrifugation at 4,424 × *g* at 4°C for 30 min. The obtained cell pellets were washed twice in ice-cold distilled H_2_O (200 mL and 50 mL, respectively) and stored at −20°C. Cell wall-associated polysaccharides (CWPSs) were extracted from cell pellets, purified as described previously (13), and further purified by fractionation on a Sephadex G-50 column (2.6 by 90 cm). Deacetylation/deamination of the CWPS of WT strain UCCSt50, monosaccharide and methylation analyses, and NMR studies were carried out as described previously (13).

### Data availability.

The genome sequences of S. thermophilus UCCSt50 (deposited under strain name S. thermophilus 4078 [5]), B1, B2, B4, and B9 were deposited in GenBank, and their associated accession numbers are as follows: CP065477, JAGSSU000000000, JAGTUD000000000, JAGSTA000000000, and JAGSSZ000000000.
