# Supplementary material for: Brussowvirus SW13 Requires a Cell Surface-Associated Polysaccharide To Recognize Its Streptococcus thermophilus Host
Source: Appl Environ Microbiol. 2022 Jan 11;88(1):e01723-21. doi: 10.1128/AEM.01723-21 (PMC8752142; doi:10.1128/AEM.01723-21)

Table S1: List of strains, plasmids and bacteriophage used in this study.

Strains	Relevance
S. *thermophilus* UCCSt50	Host strain for phage SW13
S. thermophilus UCCSt50::pNZ44-acrIIA6	Host strain harbouring the control vector
S. *thermophilus* B1	Phage insensitive derivative of *S. thermophilus* UCCSt50
S. *thermophilus* B2	Phage insensitive derivative of *S. thermophilus* UCCSt50
S. *thermophilus* B4	Phage insensitive derivative of *S. thermophilus* UCCSt50
S. *thermophilus* B9	Phage insensitive derivative of *S. thermophilus* UCCSt50
S. *thermophilus* B1::pNZ44-acrIIA6	*S. thermophilus* B1 harbouring the control plasmid
S. *thermophilus* B1::pNZ44-06955	*S. thermophilus* B1 harbouring the complementing plasmid
S. *thermophilus* B1ΔpNZ44-06955	Cured derivative of the complementing strain
**Plasmids**	
pNZ44-acrIIA6	Control vector - pNZ44 harbouring an anti-CRISPR homolog
pNZ44-06955	pNZ44-acrIIA6 harbouring native *orf*06955 from UCCSt50
pHTP9 (NZYTech, Portugal)	Gfp fusion vector used to produce recombinant Gfp-RBP-module$_{SW13}$
**Phage**	
SW13 (Accession number MH892362)	*Brussowvirus* of *S. thermophilus* UCCSt50 used for BIM generation and subsequent assays

Table S2: List of primers used in this study. Restriction sites are highlighted by bold text. The TEV cleavage site incorporated into the Gfp fusion CBD is underlined.

Primer	Sequence 5' – 3'
06955F	aaaaaa**ctgcag**aggaggtcaccttgagagttttaatgataattc
06955R	aaaaaa**actagt**ttattcactctccttcataaag
RBP-module F	tcagcaagggctgagggaaaacctgtattttcagggcagtaataacggtggtcc
RBP-module R	tcagcggaagctgaggttatatgtcttcaattgc
pNZ44F	ctaatgtcactaacctgccccg
pNZ44R	gctttatcaactgctgct

Table S3: Detail of the CRISPR arrays of the parent strain UCCSt50 and its four derived BIMs. Whilst no spacer acquisition against phage SW13 was observed, modifications were noted in the CRISPR3 array including deletions and single spacer uptake with 100% identity (Blastn) to *L. lactis* derived cloning vectors.

Strain	C1	C2	C3	Var	Significance
S. thermophilus UCCSt50	36	1	20	N/A	N /A
S. thermophilus B1	36	1	20	0	N /A
S. thermophilus B2	36	1	21	+1	100% identity to *L. lactis* derived cloning vectors
S. thermophilus B4	36	1	17	-3	Deletion of 3 spacers present in the parent strain
S. thermophilus B9	36	1	18	-3 / +1	100% identity *L. lactis* derived cloning vectors

Table S4. NMR data for *S. thermophilus* UCCSt50 Rgp and its derivatives (600 MHz, 25 °C, δ ppm).

Sugar		H/C 1	H/C 2	H/C 3	H/C 4	H/C 5	H/C 6
α-Rha A DPS	H	5.09	4.10	3.90	3.50	3.79	1.30
	C	100.9	78.0	70.7	73.2	70.7	17.7
α-Glc B DPS	H	5.01	3.55	3.76	3.47	4.16	3.70; 3.94
	C	99.1	72.4	73.9	70.7	72.0	67.5
α-Rha C DPS	H	4.93	4.03	3.92	3.47	3.74	1.30
	C	100.1	79.9	71.0	73.3	70.0	17.7
β-Gal D OS	H	4.66	3.54	3.67	3.92	3.67	3.76; 3.79
	C	104.7	72.7	73.9	69.7	76.3	62.0
α-Rha E OS	H	4.95	4.09	3.80	3.45	3.75	1.27
	C	103.3	71.1	71.1	73.1	70.3	17.7
α-Rha F OS	H	5.07	3.95	4.11	3.75	3.93	1.37
	C	99.1	79.6	71.0	81.7	68.8	18.0
anhMan G OS	H	5.09	3.83	4.20	4.17	3.95	3.68; 3.77
	C	90.5	84.9	84.8	76.6	84.2	61.6
α-Rha A PS	H	5.07	4.28	3.97	3.58	3.80	1.32
	C	100.7	77.7	79.4	72.7	71.0	17.9
α-Glc B PS	H	4.98	3.51	3.71	3.58	4.20	3.94; 3.94
	C	99.1	72.6	74.0	70.1	71.5	67.3
α-Rha C PS	H	4.93	4.06	3.92	3.48	3.75	1.34
	C	100.8	79.4	71.3	73.4	70.1	18.1
β-Gal D PS	H	4.64	3.54	3.66	3.92	3.67	3.77; 3.77
	C	104.9	72.9	74.0	69.7	76.3	62.1
α-Rha E PS	H	4.86	4.07	3.78	3.44	3.75	1.29
	C	103.7	71.3	71.3	73.3	70.1	18.1
α-Rha F PS	H	5.08	3.79	3.85	3.72	4.08	1.32
	C	100.7	81.4	71.0	81.9	68.8	17.9
β-GlcNAc G PS	H	4.76	3.73	3.63	3.47	3.47	3.76; 3.96
	C	103.6	57.0	82.7	70.2	76.9	62.1

Fig. S1 ^{1}H-^{13}C HSQC spectrum of the *S. thermophilus* UCCSt50 Rgp.

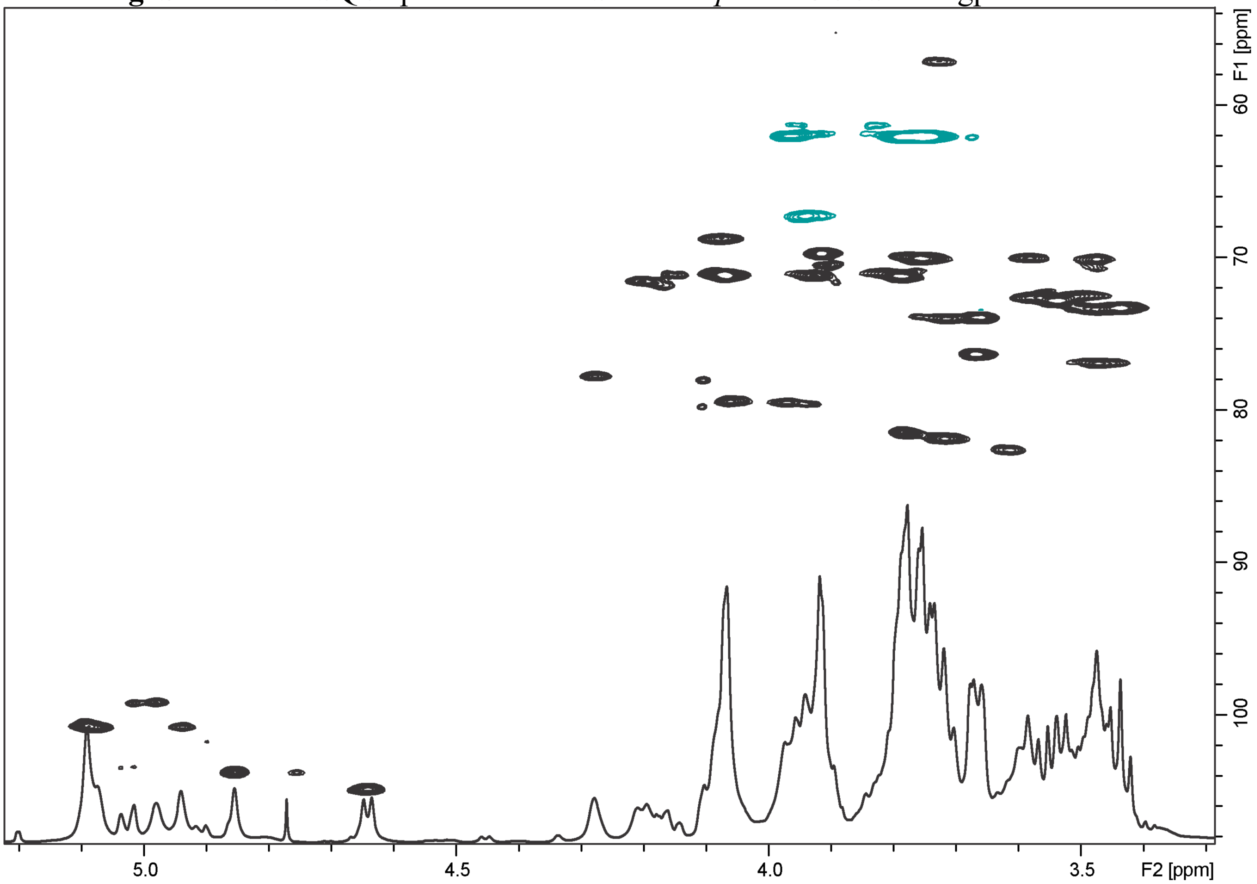

Fig. S2 ^{1}H-^{13}C HSQC spectrum of the Rgp from *S. thermophilus* UCCSt50 B1mutant.

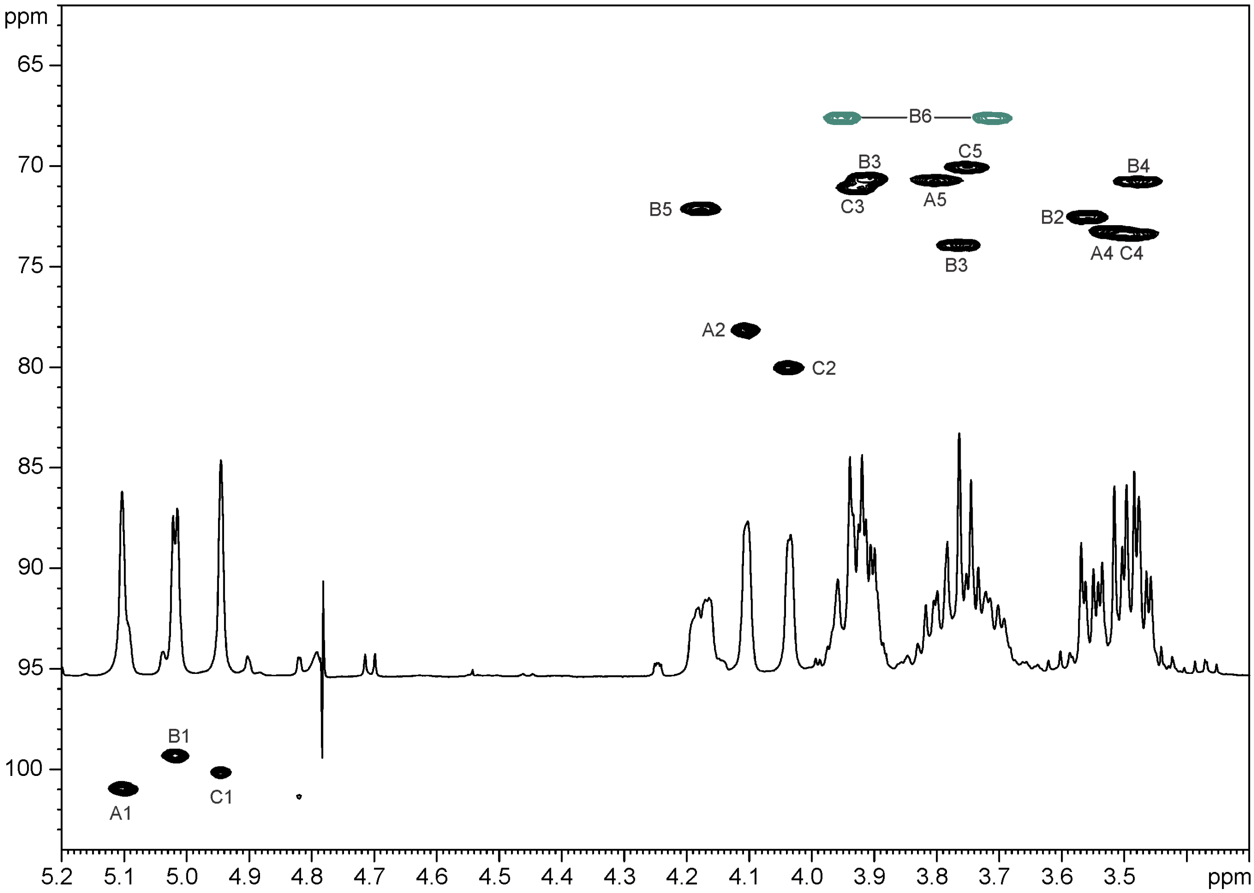

Supplement: Supplemental file 1 — Tables S1 to S4, Fig. S1 and S2. Download AEM.01723-21-s0001.pdf, PDF file, 0.3 MB [file aem.01723-21-s0001.pdf]
